# What Factors Facilitate Good Learning Experiences in Clinical Studies in Nursing: Bachelor Students' Perceptions

**DOI:** 10.1155/2013/628679

**Published:** 2013-12-17

**Authors:** Bjørg Dale, Arne Leland, Jan Gunnar Dale

**Affiliations:** Faculty of Health and Sport Sciences, University of Agder, Postboks 509, 4898 Grimstad, Norway

## Abstract

Clinical studies constitute 50% of the bachelor program in nursing education in Norway, and the quality of these studies may be decisive for the students' opportunities to learn and develop their professional competences. The aim of this study was to explore what bachelor students' in nursing perceived to be important for having good learning experiences in clinical studies. Data was collected in a focus group interview with eight nursing students who were in the last year of the educational program. The interview was transcribed verbatim, and qualitative content analysis was used for exploring and interpreting the content of the interview text. One main theme emerged from the analysis: “being in a vulnerable and exposed position characterized by conflicting needs.” Four categories were found: “aspects related to the clinical setting”, “aspects related to the nurse supervisor,” “aspects related to the student,” and “aspects related to the student-supervisor relationship”. The findings revealed that the students' learning experiences and motivation were related to individual, relational, and organizational aspects. The students highlighted their own as well as their supervisors' attitudes and competences and the importance of positive relationships. In addition, feeling welcomed, included, and valued in the ward improved their motivation, self-confidence, and self-respect.

## 1. Introduction

Organizing a good learning arena in clinical settings is crucial for bachelor students in nursing because a major part of their studies and training take place in that context. The quality of the clinical studies has, therefore, a great impact on the quality of the studies as a whole. Our impression is that the students' experiences and how they perceive the quality of their clinical studies vary a lot.

Clinical studies in nursing education are reported to represent the most stressful parts for the students, particularly in the initial periods due to lacking competence and knowledge [1, 2]. A decisive aspect for having positive learning experiences is described to be the relationship between the student and the nurse supervisor [1, 3–7]. Regular feedback, reflections, and practical advice from the supervisor are important factors for improving the students' practical competence, confidence, motivation, and self-esteem [8]. The nurses may represent a positive role figure for the students in clinical training [3]. Having regular reflections and dialogue with their clinical supervisors are, therefore, among the most important ways for the students to learn [9]. Despite this, it has been shown that the nurses' awareness of the importance of such dialogues may be absent [9]. Furthermore, this relationship may also make the students vulnerable because they are dependent on the supervisors' assessment and judgment [7].

Webb and Shakespeare [4], describe that students often strive to convince the supervisors that they are “good students” in terms of attitudes as clinical competence.

Recently, a new standardized evaluation form was introduced at the university where the current study was conducted. The evaluation form includes assessment of the nursing students' competences in hospital studies related to knowledge, skills, and attitudes. At the same time, the students' responsibility for self-evaluation is more emphasized compared with previous procedures. They need to assess their level of competence and fill out the form, and the supervisors perform their own assessment correspondently. These assessments together constitute a basis for discussion and conversation in the evaluation meeting conducted with the university teacher present. Preliminary experiences and informal feedback from students indicate that the new assessment procedure may affect several aspects related to the students' clinical training in general and the student-supervisor relationship. Therefore, the aim of the present study was to explore what bachelor students' in nursing perceived to be important for having good learning experiences in clinical studies in hospital wards after a new evaluation form was introduced.

In the presentation of the present study, the terms “supervisor” and “mentor” will be used interchangeably, and refer to the ward nurses who are responsible for supervising and assessing the students in their clinical studies. These nurses have responsibility for the student guidance and patient care at the same time; that is, they are “working nurse mentors.”

## 2. Materials and Methods

### 2.1. Participants and Data Collection

The data was collected by the second and last authors in a focus group interview performed with a group of students who were in the final year of their education. Focus group interview is described to be an appropriate qualitative data collection method for exploring experiences, attitudes, and viewpoints regarding phenomena that are common in a group of informants [10]. A purposeful sampling method was used in the recruitment of the participants [10]. Seven students who had completed their clinical studies in medical and surgical wards at the local public hospital were invited and agreed to participate in the study. It was emphasized to recruit participants who ranged in age and had experiences from different hospital wards. In addition, it was desirable that both sexes were represented. Seven students with an age ranging from 22 to 37 years constituted the group. One of the participants was male. Their previous experiences with health care varied from no experience at all to several years of experience as licensed practical nurse. A semistructured interview guide directed the interview, containing six questions about what they empathized for having good learning experiences in the clinical settings. The questions were particularly concerned with the student-supervisor relationships. The audiotaped interview lasted for one hour, and it was transcribed verbatim.

### 2.2. Data Analysis

Qualitative content analysis was used for exploring and interpreting the content of the interview text in this study. Graneheim and Lundman [11] describe this method to include both manifest and latent content of a text. The manifest content analysis concerns the description of the text as it is expressed by the informant and includes meaning units in the original text, condensation of the meaning units, and the creation of common categories. The latent content refers to the interpretation of the text and includes, according to Graneheim and Lundman [11], creation of the themes which emerge as an underlying meaning of the condensed meaning units and categories. The text was read and reread several times, moving backwards and forwards between the whole and the parts, to grasp the meaning of the phenomenon that was investigated. All authors were involved in the analysis.

In the analysis, the transcribed interview text was broken down to condensed meaning units, to grasp the manifest content of the message. In this stage, the meaning units were expressed in a language close to the interviewees' original articulations. Subsequently, the condensed meaning units were categorized, and further gathered into a main theme representing the latent content, which refers to the underlying meaning or what the text really talks about.

### 2.3. Ethical Considerations

The Norwegian Social Science Data Service (NSD) was consulted for advice regarding the obligation to report the study. They confirmed that reporting was not necessary if all personal identifying information was removed from the data set and the audiotaped interview was not downloaded on a computer. We acted in accordance with this advice and the interview was transcribed directly from the tape recorder.

In addition to the written informed consent, information about the study was repeated, and voluntariness and confidentiality were assured regarding the informants legal rights prior to each interviews [12]. Their right to withdraw at any time in the research process until publication of the study was also assured before the interview was performed.

## 3. Findings

One main theme, four categories and nine subcategories emerged from the analyses of the interview text. An overview is shown in Table 1.

In the following, the categories with their respective subcategories are presented, and some examples from the interview text are used to demonstrate the findings.

### 3.1. Aspects Related to the Clinical Setting

#### 3.1.1. Feeling Welcomed and Waited

Many of the informants emphasized the importance of being waited and feeling welcomed in the clinical ward for having a positive start. Some of them had experienced being met with an attitude characterized by surprise, unpreparedness, or even negative reactions the first day in the ward. In this sense, an important prerequisite was that the nurse leader and the staff in general were sufficiently and appropriately informed about the actual practice period, which means that information sent from the university was picked up. Knowing the students' name and educational level was important and made them feel welcomed, and, particularly, that the supervisor was informed in advance.

#### 3.1.2. A Student-Friendly Attitude and Atmosphere

While the former subcategory was mainly related to the first day or initial phase of their practice period, the informants also talked about how important it was that the ward in general had a student-friendly atmosphere. Although they appreciated to be included as a member of the staff, it was important that they were allowed to be students and use some time for their own reflections and studies. Some of them raised the problem of being (mis)used as labor resource and at the same time being in a vulnerable position regarding sanctions if they complained or avoided to do what they were told. The “evaluation ghost” was constantly present. In addition, the advantage to have two supplemental nurse supervisors was emphasized, mainly for having back-up for holidays or sick leaves. Although a lack of continuity and divergence could be a problem when having two supervisors, it was mostly pointed out as an advantage. Another important concern related to the ward was the prioritizing of time and facilities for student supervision and reflections. Although this was mainly the supervisor's responsibility, it was important that the ward and the ward manager emphasized and arranged for it.

### 3.2. Aspects Related to the Nurse Supervisor

#### 3.2.1. The Supervisor's Preparedness and Expectations

Some of the informants talked about difficult situations and experiences regarding the supervisor's lack of information and preparedness before they started the practice studies. Some of them were limited or not at all updated about the curricula or other important documents delivered from the university, for example, the composition of actual assessment form and how to make appropriate judgment of the individual student. Quite often they were not informed about the educational level of the actual student group, and they had not made themselves familiar with the students' names. Such details could influence the students' well-being and motivation.

#### 3.2.2. The Supervisor's Motivations and Attitude

The majority of the students highlighted the importance of the supervisor's motivation and their engagement regarding the mentorship. One of the informants said that

*“When I came to the ward I was allocated to a nurse who clearly expressed that she had asked to be released from the supervision duties several times-without success. She clearly expressed in many ways that that she felt discomfort and considered me as a burden”*

Another informant put it this way:

*“…they (the supervisors) always receive a folder from the university with written information and documents, but I believe that less than 20% of them read it. We experience all the time that they say that they have not been prepared or informed about the mentorship-and that they do not want it. They are obviously not motivated at all…”*

The supervisor's ability to be open, friendly, including, and willing to adjust if necessary were qualifications also mentioned by the students. Positive experiences improved their own motivation for learning and their self-confidence, and, on the contrary, difficult or bad situations could result in a negative perception of the clinical studies as a whole. One informant told about a comment she had caught from a newly graduated nurse in the ward: “… and then I heard she (the nurse) said that she looked forward to be a supervisor now so she could get her own back …” Another interviewee responded to that experience in this way: “… so I think … soon I will be a nurse myself, and then I will like to be a supervisor, and I will never treat the students the way they (the supervisors) treated me.”

#### 3.2.3. The Supervisor's Competence

When the students talked about the supervisor's competences they referred to professional competence as well as pedagogical skills. They underlined the significance of professional nursing qualifications as a decisive prerequisite for being a good mentor and a good role model. In this sense, they narrated positive as well as negative experiences. Some of the informants told about positive experiences when their supervisor prioritized to have regular appointments for supervising and time for reflections. They appreciated when the mentors challenged them and in general they felt flattered when the nurses trusted them for performing challenging tasks. However, feeling the safety of an companying mentor was expressed as one of the most important aspects for learning and developing in clinical studies. Other experiences included frustrations about the supervisors' lack of interest for updating own knowledge, and how they went in the defence if their skills were challenged. This was particularly true of the nurses who were trained years ago. One of the informants expressed:

*“Many of them (the nurses) are narrow-minded! What they learned twenty-five years ago is still the right thing for them. What we (the students) learn today … this you can just not give a damn”*

In addition to professional nursing competences the informants talked just as much about the nurses' pedagogical or supervising competences. They were critical about the lack of pedagogical skills requirement and that the allocation of mentors seemed to happen rather by chance. Some informants underlined how important it is that clinical mentors have some pedagogical education or training. One of them expressed:

*“It should be a mandatory demand from the school that all clinical supervisors should complete some kind of supervision training. Does the school really know how variable and coincidental the mentors' competences are?”*

Some of the informants expressed that they had positive experiences of having two mentors, and the advantages of how they could complement each other. This was viewed as a safety net if one of the mentors was absent for holidays, day offs, or sick leaves. Other aspects were the benefits from more than one mentor's competences and experiences, and it could work as an additional protection if the chemistry between the student and the mentor was not right.

### 3.3. Aspects Related to the Student

#### 3.3.1. The Student's Expectations and Readiness to Learn

The students' own preparedness and expectations regarding focus and learning outcomes in the actual practice period was mentioned by some of the students as an important assumption for having optimal learning benefits during the clinical studies. Furthermore, to clarify and formulate their expectations to themselves as well as to their mentors from the very start of the practice period, preferably in a written document, was clearly viewed as an advantage. One of them said:

*“It was very important for me to write down what I expected of myself and the supervisor—and what I expected from the academic teacher. I think that it is important that my supervisors know what the best way to learn for me is.”*

Another informant said:

*“I told them what I expected, and then I said that I like to be independent and take responsibilities for my own learning, so I hope that you will show me confidence and facilitate independence—within certain limits, of course. So having an open conversation from the very start about my expectations is important.”*

They acknowledged that having success regarding the achievement of the learning outcomes was largely dependent on their own efforts, engagement, curiosity, and willingness to seek and attend new learning situations. Therefore, they clearly expressed their own responsibility for having positive learning experiences.

#### 3.3.2. The Student's Competence and Confidence Level

The students' professional background and former practice experiences influenced their actual level of competence as well as the degree of self-confidence. Some of them shared stories about situations where they had been shown confidence in challenging patient situation, and how these experiences had influenced their personal and professional growth. Although information about their professional background before they started the university studies were registered in the present study, it is not unusual that nursing students have several years of former working experiences as health personnel. On the other hand, some of the students told stories about situations where they had been thrown into stressful and challenging situations without feeling comfortable and ready for it. If they failed in such situations, it could easily influence their courage and self-confidence in similar situations later. Consequently, the balance between their actual competences and experience, and their need for further instructions and training, appeared to vary a lot. Openness about their individual competences and guidance needs seemed to be a decisive factor in this sense.

Making regular diary and reflection notes were mentioned as very helpful and important tools to drive awareness of and formulate their own level of competence.

### 3.4. The Student-Mentor Relationship

#### 3.4.1. Mutual Respect and Trust

An evident thread through the entire interview was the importance of the relationship between the students and their mentors. The mutual respect included the students' understanding and respect regarding the nurses' need to share out their time and to first and foremost prioritize patient care. On the other side, it also involved the nurses' understanding and respect for the student's need for instruction and for being included, seen, and heard. Making regular appointments for reflection and feedback improved the mutual understanding, and prevented unwanted and unpredictable situations. Several stories were told about uncomfortable, unexpected, and threatening evaluation episodes caused by the lack of continuous feedback, discrepant understanding regarding the evaluation form, and disagreement about the students actual competence level. They also told about tough experiences related to an imbalanced relationship where the mentor had used their power in an unfavorable way, like some kind of punishment. Such situations could result in the students' reluctance to be open about their own insecurity or to raise questions. One of the informants said:

*“I think that many students are left with bad experiences associated with their mentors, and, you know, the consequences are that you quite simply do not dare to raise any question at all. This is not good and right!”*

To assess their own competences was obviously a stressful task for many of the informants, particularly when their own opinions and self-perceptions were considerably different from the mentor's assessment. Some of the students had experienced that the mentor gave them higher marks than they had expected. When they commented on this, even with gratitude and humbleness, they had experienced comments like “ok, then we put the mark as you perceive it.” Consequently, they felt that they were punished for being humble and they blamed themselves for not being brave enough to describe what they really felt and meant. On the other side, they also expressed disappointment and skepticism in relation to the supervisor's ability to perform an accurate assessment grounded on realistic and convincing perceptions and arguments.

#### 3.4.2. An Open and Inviting Communication

According to the students, the relationship between the student and their mentor should also be characterized by a mutual openness and a constructive, respectful, and supportive dialogue. In this respect, they pointed out their own responsibility for establishing a fruitful relationship, and they viewed their own openness and attitude just as decisive as the mentor's attitude. By continually communicating and sharing their mutual experiences, good and bad, praise and criticism, they facilitated an open atmosphere. However, corrections and criticism should be given in a constructive and supportive way and demonstrate well-meaning.

The relationship should also, according to the students, be characterized by an including communication by inviting them to share their experiences, thoughts and opinions, and by appreciating them as valuable and equal staff members.

## 4. Discussion

This study aimed to explore what bachelor students' in nursing perceived to be important for having good learning experiences in clinical studies. The overall theme that recurred throughout the interview as a red line was that the students perceived that they were in a vulnerable and exposed position. They had to continuously balance their own needs for appropriate learning experiences and respect for the nurses' lack of time to provide as much guidance as they wanted. Being constantly controlled and evaluated made them vulnerable because they wanted to make a positive impression and they were reluctant to take up things that could be perceived negatively. In addition, to find the balance between taking challenges and to express uncertainty in unfamiliar situations made them vulnerable and exposed.

Almost all the informants talked about the importance of having a positive start when they started their studies in a new ward. Experiences from earlier practice periods could vary a lot and influence their expectations and confidence level, but they often felt stressed and a bit anxious before the first meeting. Thus, feeling waited and welcomed, and being met with friendliness from the very beginning, seemed to be decisive for their further motivation and confidence level. In their study, Courtney-Pratt et al. [13] found that students in general felt welcomed, belonging, and accepted in the ward. On the other hand, Sharif and Masoumi [1] describe that the initial phase of clinical placement represented an anxious and stressful phase for the students. They are particularly afraid of making mistakes when performing patient procedures, and for harming the patients because they lack experience and knowledge [1, 14].

The aspect of balancing the appreciation of being included and valued as a staff member and being (mis)used as merely a pair of hands during their clinical studies was another concern mentioned by many of the informants. This balancing act, which can often be difficult, is also outlined in other studies [5, 15]. On one side, students appreciate being valued as a team member [16], but on the other side, they may feel uncomfortable when they are used solely as work force rather than being valued as learners [5]. One factor that made this balancing act even harder was that they would be judged and evaluated. Experiences of being ignored and (mis)used as health care assistants or purely work force is shown to cause a great deal of “emotional labor” for students in clinical settings [4]. On the contrary, the feeling of being seen, heard and valued as individuals as well as students is described as an important prerequisite for experiencing good learning situations [5, 17]. In this sense, facilitating for good learning experiences does not solely rely on the mentor, but it is an organizational matter as well.

Many of the interviewees thought it was an advantage to have two supervisors who supplemented each other. One explanation to this finding may be the problems related to the mentors' absence, leaving the students being without supervision, and feedback, which also in another study is shown to cause frustration and insecurity [8]. On the other hand, studies have also shown that the allocation of students to more than one mentor has been recognized as a stressful factor for the students, and it may result in weakened feelings of belonging [13, 18]. Thus, although a risk for conflicting situations due to the mentors' different attitudes and pedagogical approach, the students may experience a copartner mentorship as favorable. The advantage of having two supervisors with different expertise and experience, and thus complement each other, is also reported by Webb and Shakespeare [4].

Several aspects related to the nurse mentor were described by the informants as crucial for their learning experiences during their clinical studies. One of these aspects was the mentors' motivation for supervising students, which also included that they were prepared and that they had acquired the necessary information about the curricula and the assessment form. The mentors' motivation for supervision is often reflected in their attitudes towards the students, and their willingness to teach them and share their knowledge with them [4, 19]. Although the students were aware of and respected that the nurses' primary duties were related to patient care, they also expected that the supervisors facilitated and allocated time for talks, reflection, and guidance. Good learning experiences in practice are shown to be clearly related to perceiving support, friendliness, and having mentors who stand by in new and stressful practice situations [3, 4]. The findings also indicate that the students' experiences of negative or bad attitudes from the mentors can result in learning. The consequence of such experiences can be that the students take an opposite stance and they condemn such attitudes when they shall have the supervisor role in the future. In this way, negative experiences may be turned into learning opportunities by deciding how not to act in similar situations [3].

In addition to being supportive and encouraging, the students also emphasized their mentors' professional and pedagogical competences. While the former is well documented in earlier studies [3, 6, 17, 20], the literature focusing on the latter is more infrequent. Webb and Shakespeare [4] who found that well experienced nurses brought a well of knowledge and skills into the mentorship, which provided the students with a lot of learning opportunities. Well experienced and trained nurses may demonstrate nursing practice of eminent quality, be exemplary role models, and facilitate the students' self-confidence when used in appropriate ways. Nurses who are comfortable with their mentor role and possess sufficient educational qualifications may also affect students' learning outcomes in positive ways. The informants in the present study expressed criticism and skepticism to the lack of supervising skills they had experienced from some of the mentors. Problems related to the nurse mentor's lack of appropriate qualifications for supervising students may affect the students' possibility to improve their clinical competences [15]. It may be that the supervisor, for example, lacks the ability and preparation to give relevant and regular feedback [8].

The students put a lot of emphasis on aspects related to the mentor and the mentor-student relationship for experiencing good learning situations, and these aspects have also been documented in former studies [3, 4, 7]. However, the informants in the current study also acknowledged their own responsibility for achieving learning outcomes. This finding is quite interesting, and earlier research focusing on this aspect as a prerequisite for good learning experiences is sparse. The students' responsibility for their own learning in clinical studies is clearly described in the framework for nursing education [21]. In this respect, clarification of their current competences, and what individual learning goals they expect to achieve and how to achieve them, should be presented in the initial phase of the clinical studies. The assessment form and evaluation procedure which are currently used also require that the students continuously make up their mind and evaluate themselves concerning different types of competences. Self-assessment skills in nursing studies are described to be a necessity for competent performance and for developing autonomous and independent practitioners [22]. The informants further described that clarification of their own competence level and learning outcomes related to each practice placement facilitated for appropriate learning activities. Opportunities for further growth are more likely present when the nurse mentors are informed about, and comfortable with, the students' professional starting point, and it may be easier to find the balance between taking challenges and being reticent [7].

The aspect most often described by the informants to be of utmost importance for their development and learning in clinical studies was the quality of the relationship that they had with their supervisors. A relationship characterized by mutual respect, both as individuals and professionals, was highly empathized. Firstly, this respect included the students' recognition of the nurses' pedagogical and professional competences as well as their huge workload. Studies performed among nurse supervisors have reported that shortage of time and insufficient skills were perceived as challenging factors related to supervising students [3, 23]. In the same way, the students wanted the nurses to respect and regard their actual competence level and their individual needs for supervision and guidance as well as their need to take on challenges. Thus, having enough time for feedback and reflections, and having the opportunities to safely practice their skills, are particularly important [4, 11]. This means that the supervisor is attending and supporting when the student is practicing new skills. It also means that the student is challenged and is allowed to take responsibility and perform nursing procedures on their own. When they experience the supervisor's confidence in such situations, the students feel valued, seen, and respected [14].

The fact that they are continuously controlled and assessed in their clinical studies makes the students feel dependent and vulnerable [7]. Therefore, a relationship characterized by an open and inviting atmosphere, where both parties are encouraged to communicate positive as well as negative feedback, should be established. Such relationships can make a difference in how confident they are when it comes to seeking advice and assistance [13]. Terms such as friendly, supportive, welcoming, enthusiastic, and confident have been used to describe the characteristics of a good student-supervisor relationship [13]. Consequently, communication and feedback carried out in a respectful, friendly, and supportive way may result in good learning experiences and growth for the students, even in problematic situations.

## 5. Study Limitations

In qualitative studies it is not appropriate to make generalizations of the findings. In general, the purpose of qualitative studies is to explore the meaning of individuals' experiences or the nature of phenomena, and a transcribed interview text may be understood and interpreted in various ways. Therefore, the present interpretation should be viewed as one among many possibilities. However, the findings contribute to existing knowledge of what nursing students' perceive as important aspects for good learning experiences in their clinical studies. The interview text provided rich descriptions of the informants' experiences, and variations and similarities in these descriptions were recognized. The study sample was limited and consisted of students in the same class at one university. Despite these limitations, many of the experiences described by the students correspond to findings in other international and national studies.

## 6. Conclusions

The findings in this study showed that important conditions for nursing students' learning experiences and motivation in their clinical studies included individual, relational, and organizational aspects. Clinical studies constitute a major part of the bachelor program in nursing and, consequently, the quality of these studies is a crucial factor for the students to develop into competent professional nurses. To clarify the students' competence level and learning outcomes, as well as the students' and supervisors' mutual expectations, responsibility, and roles, seems to be decisive for the students' opportunities for personal and professional learning and growth. Feeling welcomed, included, and valued in the ward improves the students' motivation, self-confidence, and self-respect. In addition, the quality of the student-supervisor relationship and the supervisor's pedagogical and professional competences are highly emphasized.

## Figures and Tables

**Table 1:** Overview of the main theme, categories, and associated subcategories.

Main theme			
Being in a vulnerable and exposed position characterized by conflicting needs			
Category 1	Category 2	Category 3	Category 4

Aspects related to the clinical setting	Aspects related to the nurse supervisor	Aspects related to the student	Aspect related to the student-supervisor relationship

Subcategories	Subcategories	Subcategories	Subcategories

Feeling welcomed and waited	The supervisor's preparedness and expectations	The student's expectations and readiness to learn	Mutual respect and trust
A student-friendly attitude and atmosphere	The supervisor's motivations and attitude	The student's competences and confidence level	An open and inviting communication
	The supervisor's competence		
